# Evaluating the distribution of African swine fever virus within a feed mill environment following manufacture of inoculated feed

**DOI:** 10.1371/journal.pone.0256138

**Published:** 2021-08-12

**Authors:** C. Grace Elijah, Jessie D. Trujillo, Cassandra K. Jones, Natasha N. Gaudreault, Charles R. Stark, Konner R. Cool, Chad B. Paulk, Taeyong Kwon, Jason C. Woodworth, Igor Morozov, Carmina Gallardo, Jordan T. Gebhardt, Jürgen A. Richt

**Affiliations:** 1 Department of Diagnostic Medicine/Pathobiology, College of Veterinary Medicine, Kansas State University, Manhattan, Kansas, United States of America; 2 Center of Excellence for Emerging and Zoonotic Animal Disease, Kansas State University, Manhattan, Kansas, United States of America; 3 Department of Animal Sciences and Industry, College of Agriculture, Kansas State University, Manhattan, Kansas, United States of America; 4 Department of Grain Science and Industry, College of Agriculture, Kansas State University, Manhattan, Kansas, United States of America; 5 Instituto Nacional de Investigación y Tecnología Agraria y Alimentaria, Animal Health Research Centre, Madrid, Madrid, Spain; University of Nicolaus Copernicus in Torun, POLAND

## Abstract

It is critical to understand the role feed manufacturing may have regarding potential African swine fever virus (ASFV) transmission, especially given the evidence that feed and/or ingredients may be potential vectors. The objective of the study was to evaluate the distribution of ASFV in a feed mill following manufacture of contaminated feed. To accomplish this, a pilot-scale feed mill consisting of a mixer, bucket elevator, and spouting was constructed in a BSL-3Ag facility. First, a batch of ASFV-free feed was manufactured, followed by a batch of feed that had an ASFV-contaminated ingredient added to feed, which was then mixed and discharged from the equipment. Subsequently, four additional ASFV-free batches of feed were manufactured using the same equipment. Environmental swabs from 18 locations within the BSL-3Ag room were collected after each batch of feed was discharged. The locations of the swabs were categorized into four zones: 1) feed contact surface, 2) non-feed contact surface < 1 meter away from feed, 3) non-feed contact surface > 1 meter from feed, and 4) transient surfaces. Environmental swabs were analyzed using a qPCR specific for the ASFV p72 gene and reported as genomic copy number (CN)/mL of environmental swab processing buffer. Genomic copies were transformed with a log_10_ function for statistical analysis. There was no evidence of a zone × batch interaction for log_10_ genomic CN/mL (*P* = 0.625) or cycle threshold (Ct) value (*P* = 0.608). Sampling zone impacted the log_10_ p72 genomic CN/mL (*P* < 0.0001) and Ct values (*P* < 0.0001), with a greater amount of viral genome detected on transient surfaces compared to other surfaces (*P* < 0.05). This study illustrates that once ASFV enters the feed mill environment it becomes widespread and movement of people can significantly contribute to the spread of ASFV in a feed mill environment.

## Introduction

Commercial swine feed serving as a fomite for transmission of viral pathogens was not deemed a significant concern until soon after diagnosing porcine epidemic diarrhea virus (PEDV) in the US in 2013. It was reported that contaminated feedstuffs or their packaging arriving from Asia may have been involved with the introduction and transmission of PEDV in North America [1]. Due to the US naïve status to PEDV at the time along with the movement of contaminated vehicles associated with feed and animal delivery, the virus became endemic in the US. Another contributing factor to the quick spread of PEDV in the US was the feed mill. Once introduced into the feed mill, PEDV became widely distributed [2], serving as a continuous source of disease to the workers and feed delivery vehicles. Decontamination methods were often unsuccessful to rid the environment of PEDV but were also expensive and time consuming [3] while sequencing of diets within the feed mill to dilute the virus within the feed were also unsuccessful at eliminating PEDV [4]. The outbreak of PEDV in the US was the first to suggest that the feed manufacturing and distribution system aided in the widespread transmission of disease.

African swine fever is a devastating virus endemic in Africa, Asia, and Europe with substantial impacts on swine production and economic implications [5]. Multiple routes of ASFV transmission to domestic swine have been characterized including domestic swine to domestic swine, wild suids to domestic swine, and via the soft tick of the *Ornithodoros* genus serving as a vector [6]. Within these, multiple mechanisms of transmission can occur whereby domesticated swine become infected with ASFV including direct animal contact, contact with contaminated fomites, or exposure to contaminated feedstuffs or water [7]. Extended stability of ASFV within pork products has been widely documented [8], detection of ASFV DNA within pork products crossing international borders has been documented [9, 10], and strong evidence of ASFV transmission via contaminated food products has been documented [11, 12].

Given the known fact that contaminated pork products can lead to ASFV infection in naïve animals, recent research has focused on further understanding of the risk of commercial swine feed serving as a vector for ASFV transmission. Recent research has shown that ASFV can survive in various feed ingredients during transboundary, transatlantic shipping and work has been conducted characterizing the infectious dose in water and feed [13, 14]. Field evidence suggests that ASFV can be distributed throughout the feed supply chain [15], but there are no controlled studies demonstrating the properties of ASFV in a feed mill environment, making it impossible to develop science-based recommendations or policy. Therefore, the objective of this study was to use an ASFV-contaminated ingredient in the feed manufacturing process to evaluate the cross-contamination to subsequent batches of feed and contamination of the feed mill environment.

## Materials and methods

The study was conducted at the Biosecurity Research Institute (BRI) in Manhattan, KS, with approval by the Kansas State University Institutional Biosafety Committee (project approval #1427.1). The feed manufacturing process was done within a BSL-3Ag large animal room while laboratory work was done within a BSL-3+ laboratory space.

### Preparation of the inoculum

A total 8.5 mL of pooled blood treated with ethylendiaminetetraacetic acid (EDTA) from ASFV infected pigs was mixed in RPMI media to prepare 530 mL of the virus inoculum at the final concentration of 2.7 × 10^6^ TCID_50_/mL of ASFV genotype II virus (Armenia 2007).

### Feed manufacturing

Feed was manufactured as described by Schumacher et al. [2]. Briefly, the feed manufacturing system was first primed with an ASFV-free batch of feed which was subsequently followed by a second batch of feed that was contaminated with ASFV. Four additional batches of ASFV-free feed were then mixed and discharged through the same equipment without any cleaning or disinfection occurring between batches. For this study, a corn and soybean-meal based diet with a composition normally fed to gestating sows was manufactured at the Kansas State University O.H. Kruse Food Technology Innovation Center (Manhattan, KS; Table 1) and transported to the BSL-3Ag facility.

**Table 1:** Diet composition (as-fed basis).

Item	Swine gestation diet
Ingredient, %	
Corn	78.41
Soybean meal^1^	17.27
Soybean oil	0.50
Calcium carbonate	1.30
Monocalcium phosphate	1.30
Sodium chloride	0.50
Trace mineral^2^	0.15
Sow add pack^3^	0.25
Vitamin premix^4^	0.25
Phytase^5^	0.08
Total	100
Calculated analysis, %^6^	
Crude protein	14.7
Crude fiber	3.5
Crude fat	2.2
Total calcium	0.91
Total phosphorous	0.61

^1^ Conventional dehulled, solvent extracted soybean meal.

^2^ Each kg of premix contains 73 g Fe, 73 g Zn, 22 g Mn, 11 g Cu, 198 mg I, and 198 mg Se.

^3^ Each kg of premix contains 1,650,000 IU vitamin A, 8,800 IU vitamin E, 88 mg biotin, 396 mg pyridoxine, 880 mg folic acid, 220,000 mg choline, 79 mg chromium, 19,800 mg L-carnitine.

^4^ Each kg of premix contains 1,650,000 IU vitamin A, 660,000 IU vitamin D3, 17,600 IU vitamin E, 1,320 mg menadione, 3,300 mg riboflavin, 11,000 mg d-pantothenic acid, 19,800 mg niacin, 13 mg vitamin B12.

^5^ HiPhos 2700 (DSM Nutritional Products, Parsippany, NJ).

^6^ NRC. 2012. Nutrient Requirements of Swine, 11th ed. Natl. Acad. Press, Washington D.C.

#### Negative control (Batch 1)—Priming the feed mill

To initiate the trial, a 25 kg batch of ASFV-free feed was mixed in a 50 kg capacity steel mixer with a 0.113 m^3^ electric paddle mixer (H.C Davis Sons Manufacturing, model # SS-L1; Bonner Springs, KS). The feed was mixed for five minutes then discharged at a rate of approximately 4.5 kg/min into the conveyor (Universal Industries, Cedar Falls, IA) that carried 74 buckets (each 114 cm^3^) of feed. The feed was conveyed and discharged through a downspout into double-lined bags.

#### Positive control (Batch 2)—ASFV-contaminated feed

Upon completion of priming the system with the initial batch of ASFV-free feed, 530 mL of a genotype II (Armenia 2007) ASFV (2.7 × 10^6^ TCID_50_/mL) was then mixed with 4.7 kg of diet in a 5 kg stainless steel mixer (Cabela’s Inc., Sidney, NE) to make 5.23 kg of ASFV-contaminated feed. This was subsequently added to 20 kg of feed and then mixed, conveyed, and discharged using the same equipment and procedures as previously described for the negative control. The final concentration of the inoculated positive control batch of feed was 5.6 × 10^4^ TCID_50_/gram.

#### Sequences 1–4 (Batch 3, 4, 5, and 6)—Milling of subsequent batches of feed

Following discharge of the positive control batch of feed, the same process of mixing, conveying, and discharging 25 kg batches of feed was repeated four additional times using ASFV-free diet.

### Environmental sampling

Environmental sampling was conducted similar to Huss et al. [3] and Schumacher et al. [2]. Environmental swabs were taken for the negative control, positive control, and batch sequences 1–4. Negative control samples were taken after priming the feed mill, positive control samples were taken after the usage of ASFV- contaminated feed, and batch sequences 1–4 samples were taken after each subsequent batch. All environmental swabs collected on previously marked environmental surfaces prior to inoculation with ASFV had no detectable ASFV DNA.

After each batch of feed was manufactured, environmental surfaces were swabbed using 10 cm × 10 cm cotton surgical gauze squares pre-moistened with 5 mL of phosphate-buffered solution (PBS) and individually stored in a 50 mL conical tube prior to usage. Prior to sample collection, a clean pair of outside gloves were donned and tubes aseptically opened by a sampling assistant. The previously chosen and marked location was swabbed, the environmental swab placed back in the conical tube, and outside gloves were changed. Once the experiment was concluded, samples were transferred to the BSL-3+ laboratory following appropriate procedures.

Locations for environmental sampling were chosen based off proximity to feed (Table 2). Feed contact surface locations were the mixer ribbon, mixer barrel, mixer discharge, bucket elevator bucket, bucket elevator belt, and bucket elevator discharge. Non-feed contact surfaces < 1 m from feed locations were wall less than 1 m to mixer, wall less than 1 m to bucket elevator, floor less than 1 m to mixer, floor less than 1 m to bucket elevator, and ceiling less than 1 m to mixer. Non-feed locations > 1 m from feed locations were wall greater than 1 m from mixer, floor greater than 1 m from mixer, floor greater than 1 m from bucket elevator, and ceiling greater than 1 m from mixer. Transient surface locations were the boot soles of researchers walking through all other zones.

**Table 2:** Location of environmental swabs and grouping by zone.

Zone type	Location
Feed contact surface	Mixer ribbon
	Mixer barrel
	Mixer discharge
	Bucket elevator bucket
	Bucket elevator belt
	Bucket elevator discharge
Non-feed contact surface < 1 meter away from feed contact surface	Wall close to mixer
	Wall close to bucket elevator
	Floor close to mixer
	Floor close to bucket elevator
	Ceiling close to mixer
Non-feed contact surface > 1 meter away from feed contact surface	Wall far from mixer
	Floor far from mixer
	Floor far from bucket elevator
	Ceiling far from mixer
Transient surface	Boot sole of researcher A
	Boot sole of researcher B
	Boot sole of researcher C

### DNA extraction and quantitative ASFV real-time PCR (qPCR)

Environmental swabs were tested at a BSL-3+ laboratory in the Biosecurity Research Institute in Manhattan, KS. Briefly, to each swab within a 50 mL conical tube, 20 mL of PBS was added, the tube was capped and inverted, and incubated overnight in 4°C. Tubes were vortexed for about 30 seconds and held upright for 5 minutes. Approximately 10 mL of supernatant was recovered, aliquoted into 5 mL cryovials, and stored at -80°C until processed for qPCR. In preparation for magnetic bead-based DNA extraction, 500 μL of PBS eluent was combined with 500 μL of Buffer AL (Qiagen, Germantown, MD, USA), briefly vortexed, and incubated at 70°C for 10 minutes in an oscillating heat block. DNA extraction was carried out using the GeneReach DNA/RNA extraction kit on a Taco^™^ mini automatic nucleic acid extraction system (GeneReach, Boston, MA, USA). The extraction was performed according to the manufacturer’s instructions with modifications. Briefly, 200 μL of AL sample lysate was transferred to column A of the taco deep-well extraction plate which contained 500 μL of the GeneReach lysis buffer and 50 μL of magnetic beads, followed by addition of 200 μL of molecular grade isopropanol (ThermoFisher Scientific, Waltham, MA, USA). The extraction consisted of two washes with 750 μL of wash buffer A, one wash with 750 μL wash buffer B, and a final wash with 750 μL of 200 proof molecular grade ethanol (ThermoFisher Scientific). After a five-minute drying time, DNA was eluted with 100 μL elution buffer and subsequently transferred into 1.5 mL DNA/RNA- free centrifuge tubes (VWR) for storage. Positive and negative extraction controls were included in sample processing and consist of the positive extraction control, a partial sequence of the ASFV p72 gene cloned into plasmid Bluescript II and PCR-grade water.

Real-time quantitative PCR (qPCR) was carried out using primers and probes designed to detect the gene encoding for ASFV p72 [16] and PerfeCTa^®^ FastMix II^®^ (Quanta Biosciences, Gaithersburg, MD, USA) on the CFX96 Touch^™^ Real-Time PCR Detection System (Bio-Rad, Hercules, CA, USA). qPCR reactions were performed in duplicate with each well containing 5 μL of template DNA, 0.2 μL (200nM) of each primer (Integrated DNA Technology, Coralville, IA, USA), and 0.4 μL (200nM) of FAM probe (Thermo Fisher Scientific) in a total reaction volume of 20 μL. Thermocycling conditions were 95°C for 5 minutes, followed by 45 cycles of 95°C for 10 seconds and 60°C for 1 minute.

### Genomic copies quantification

ASFV p72 genomic copy number (CN) was calculated using reference standard curve methodology using a reference standard curve composed from ten-fold serial dilutions performed in triplicate of the quantitated ASFV p72 plasmid DNA control. Copy number for samples were mathematically determined using the PCR-determined cycle threshold (Ct) for ASFV p72 (two PCR well replicates) and the slope and intercept of the ASFV p72 DNA standard curve. Data are reported as PCR determined copy number per mL of solution recovered from environmental swab sample processing.

### Statistical analysis

Data were analyzed as 4 × 5 factorial arrangement with 4 sampling surfaces, and 5 batches of feed not including the initial negative control samples. Individual sample collected from a surface for a specific batch was considered the experimental unit.

Visualization on data was performed using the ggplot2 package using the RStudio environment (Version 1.2.1335, RStudio, Inc., Boston, MA) using R programming language [Version 3.6.1 (2019-07-05), R Core Team, R Foundation for Statistical Computing, Vienna, Austria]. The proportion of PCR reactions positive for detectable ASFV DNA are reported as # of PCR positive reactions/total # of PCR reactions. The proportion of PCR reactions having detectable ASFV DNA was fit using the glmer function in the lme4 package using a binomial distribution with the fixed effects of sampling zone, batch of feed, and the associated interaction with a random effect of environmental swab to indicate the appropriate level of experimental replication given the duplicate qPCR analysis of environmental swabs.

Genomic CN/mL and Ct were analyzed using a linear mixed model fit using the lme function in the nlme package using similar fixed and random effects as previously mentioned. Results of Ct and p72 genomic CN/mL data are reported as least squares means ± standard error of the mean. Samples not containing detectable ASFV DNA were assigned a value of 45 because that was the greatest number of cycles the qPCR assay performed before concluding a sample did not have detectable ASFV DNA. Genomic CN/mL values were log_10_ transformed prior to data analysis to satisfy the assumption of normality. All statistical models were evaluated using visual assessment of studentized residuals and models accounting for heterogeneous residual variance were used when appropriate. A Tukey multiple comparison adjustment was incorporated when appropriate. Results were considered significant at *P* ≤ 0.05 and marginally significant between *P* > 0.05 and *P* ≤ 0.10.

## Results

As expected, environmental swabs collected prior to inoculation had no detectable ASFV DNA (Table 3). Environmental swabs collected after the manufacture of ASFV-contaminated feed showed presence of ASFV-specific DNA in all zones with 38% (95% confidence limit = 6.4–78.3%) to 100% (95% confidence limit = 0–100%) of qPCR reactions resulting in detectable ASFV DNA depending on the contact surface. There was no evidence of a sampling zone × batch of feed interaction for prevalence of qPCR reactions detecting ASFV DNA (*P* = 0.912), log_10_ genomic copies/mL (*P* = 0.625), or Ct value (*P* = 0.608). Additionally, there was insufficient evidence to conclude that the proportion of qPCR positive reactions was affected by sampling zone (*P* = 0.701) or batch of feed (*P* = 1.000).

**Table 3:** Interactive effect of feed batch and zone on detection of African swine fever virus (ASFV) during manufacture of virus inoculated feed^1^^,^^2^.

	Batch of feed					
Item	Negative	Positive	After sequence 1	After sequence 2	After sequence 3	After sequence 4
Detectable DNA/Total^3^						
Feed contact	0/12	9/12	6/12	5/12	6/12	5/12
Non-feed contact, < 1 m	0/10	8/10	5/10	4/10	1/10	3/10
Non-feed contact, > 1 m	0/8	3/8	4/8	4/8	3/8	3/8
Transient surface	0/6	6/6	6/6	6/6	6/6	6/6
Log_10_ genomic copy number/mL^4^						
Feed contact	0	2.74 ± 0.481	1.51 ± 0.481	1.16 ± 0.481	1.75 ± 0.481	1.32 ± 0.481
Non-feed contact, < 1 m	0	2.70 ± 0.526	1.55 ± 0.526	1.04 ± 0.526	0.28 ± 0.526	0.86 ± 0.526
Non-feed contact, > 1 m	0	0.96 ± 0.589	1.27 ± 0.589	1.45 ± 0.589	0.91 ± 0.589	1.06 ± 0.589
Transient surface	0	4.44 ± 0.455	4.07 ± 0.455	3.92 ± 0.455	3.83 ± 0.455	4.14 ± 0.455
Cycle threshold^5^						
Feed contact	45.0	37.3 ± 1.33	41.1 ± 1.33	42.2 ± 1.33	40.2 ± 1.33	41.5 ± 1.33
Non-feed contact, < 1 m	45.0	37.7 ± 1.46	41.0 ± 1.46	42.8 ± 1.46	44.3 ± 1.46	42.9 ± 1.46
Non-feed contact, > 1 m	45.0	42.8 ± 1.63	42.3 ± 1.63	41.4 ± 1.63	43.0 ± 1.63	42.4 ± 1.63
Transient surface	45.0	31.6 ± 1.40	33.1 ± 1.40	33.7 ± 1.40	34.1 ± 1.40	32.8 ± 1.40

^1^ Swine gestation feed was inoculated with African swine fever virus (ASFV) at 5.6 × 10^4^ TCID_50_/gram inoculated feed (positive) following an initial priming of the feed manufacturing equipment with ASFV free feed (negative). Four subsequent batches of feed were manufactured (sequence 1 to 4) and were initially free of ASFV. Environmental samples were collected at multiple locations within the facility following each batch of feed and were analyzed using an ASFV p72 encoding gene qPCR assay.

^2^ Statistical analysis includes all treatment groups except for negative control.

^3^ Count of PCR reactions with detectible ASFV DNA/number of qPCR reactions for each combination of sampling location and batch with each sampling swab was analyzed by duplicate reactions; Zone × Batch, *P* = 0.912; Zone, *P* = 0.701; Batch, *P* = 1.000.

^4^ Log_10_ transformed genomic copies for ASFV p72 encoding gene per mL of solution recovered from environmental swab sample ± standard error of mean. Zone × Batch, *P* = 0.625; Zone, *P* < 0.0001; Batch, *P* = 0.059.

^5^ Cycle threshold values with samples having no detectable ASFV DNA (ND) being assigned a value of 45 within the statistical analysis ± standard error of mean. Zone × Batch, *P* = 0.608; Zone, *P* < 0.0001; Batch, *P* = 0.037.

Batch of feed influenced the Ct value for environmental samples (*P* = 0.037), with samples collected after manufacture of the ASFV- contaminated batch of feed having a lower Ct value compared to the environmental swabs collected after sequence 3 (*P* < 0.05; Table 4). Environmental swabs collected after other sequences (1, 2, 4) were intermediate in terms of Ct value. There was marginally significant evidence that batch of feed influenced log_10_ p72 genomic copy/mL (*P* = 0.059), however no significant pairwise differences were detected when using a Tukey multiple comparison adjustment.

**Table 4:** Main effect of feed batch and zone on detection of African swine fever virus (ASFV) during manufacture of virus inoculated feed^1^^,^^2^.

Main effect	Detectable DNA/Total^3^	Log_10_ genomic copy number/mL^4^	Cycle threshold^5^
Batch			
Negative	0/36	0	45.0
Positive	26/36	2.71 ± 0.258	37.4 ± 0.73^a^
After sequence 1	21/36	2.10 ± 0.258	39.4 ± 0.73^a^^,^^b^
After sequence 2	19/36	1.89 ± 0.258	40.0 ± 0.73^a^^,^^b^
After sequence 3	16/36	1.69 ± 0.258	40.4 ± 0.73^b^
After sequence 4	17/36	1.85 ± 0.258	39.9 ± 0.73^a^^,^^b^
Zone			
Feed contact	31/60	1.70 ± 0.215^a^	40.5 ± 0.60^a^
Non-feed contact, < 1 m	21/50	1.29 ± 0.235^a^	41.7 ± 0.65^a^
Non-feed contact, > 1 m	17/40	1.13 ± 0.263^a^	42.4 ± 0.73^a^
Transient surface	30/30	4.08 ± 0.203^b^	33.1 ± 0.63^b^

^1^ Swine gestation feed was inoculated with African swine fever virus (ASFV) at 5.6 × 10^4^ TCID_50_/gram inoculated feed (positive) following an initial priming of the feed manufacturing equipment with ASFV-free feed (negative). Four subsequent batches of feed were manufactured (sequence 1 to 4) and were initially free of ASFV. Environmental samples were collected at multiple locations within the facility following each batch of feed and were analyzed using an ASFV p72 encoding gene qPCR assay.

^2^ Statistical analysis includes all treatment groups except for negative control where samples were collected prior to ASFV inoculation. Values for main effect of contact surface do not include negative batch of feed.

^3^ Count of PCR reactions with detectible ASFV DNA/number of qPCR reactions for each combination of sampling location and batch with each sampling swab was analyzed by duplicate reactions; Batch, *P* = 1.000; Zone, *P* = 0.701.

^4^ Log_10_ transformed genomic copies for ASFV p72 encoding gene per mL of solution recovered from environmental swab sample; Batch, *P* = 0.059; Zone, *P* < 0.0001.

^5^ Cycle threshold values with samples having no detectable ASFV DNA being assigned a value of 45 within the statistical analysis; Batch, *P* = 0.037; Zone, *P* < 0.0001.

^abc^ Means within main effect lacking common superscript differ (*P* < 0.05) using Tukey multiple comparison adjustment.

There was a significant difference in both the Ct value and log_10_ genomic copy/mL values between sampling zones (*P* < 0.0001), with the transient surfaces having lower Ct values (*P* < 0.05) and greater log_10_ p72 genomic copies/mL (*P* < 0.05) compared to all other sampling zones. This indicates that the soles of worker boots contained a greater quantity of detectable ASFV DNA compared to all other sampling zones, including feed contact and non-feed contact surfaces.

## Discussion

African swine fever is a devastating disease not only for substantial morbidity and mortality, but also serious economic consequences associated with global trade [5]. The virus is a double-stranded DNA virus of the family *Asfarviridae* and has an external lipid envelope [17]. Multiple routes of transmission to domestic swine have been characterized including domestic swine to domestic swine, wild suids to domestic swine, and soft tick of the *Ornithodoros* genus [6]. Within these, multiple mechanisms of transmission can occur whereby domesticated swine become infected with ASFV including direct animal contact, contact with contaminated fomites, or exposure to contaminated feedstuffs or water [7]. Extended stability of ASFV within pork products has been widely documented [8], detection of ASFV DNA within pork products crossing international borders has been documented [9, 10], and strong evidence of ASFV transmission via contaminated food products has been documented [11, 12]. Thus, understanding the risk for ingestion of ASFV contaminated feedstuffs is very important to prevent infection with ASFV in swine populations. While it has clearly been demonstrated that consumption of contaminated food products can result in ASFV transmission, the risk of feedstuffs serving as a potential vector for pathogen transmission in modern swine production with limited access to potentially contaminated pork products is not as well characterized to date.

Traditionally, biosecurity for the swine industry has focused on preventing pathogen entry onto the farm by controlling the safety of incoming animals, personnel, and supplies. However, the entry and spread of PEDV throughout North American in 2013–2014 speculated that feed could serve as a fomite and the feed supply chain could help spread the virus but ultimately, research demonstrated that feed could serve as a potential fomite for viral transmission and contaminate the feed delivery supply chain [1]. The introduction and dissemination of PEDV in North America served to shift the mindset of swine producers and feed manufacturers and a greater degree of attention was directed towards a framework for extending biosecurity practices to feed and feed mills. Previous research using other viruses [18–22] has established that mitigation techniques are largely expensive and impractical, so prevention of pathogen introduction into the feed supply chain is critical. Lessons learned through experiences with PEDV in regards to feed biosecurity served to shift the mindset and practices of the swine feed industry.

Previous work with PEDV has demonstrated that once viral contamination is introduced into feed manufacturing equipment, the contamination can be detected on surfaces after several subsequent batches of feed [2]. Furthermore, contamination on non-feed contact surfaces persists longer than contamination of feed-contact surfaces due to the abrasive and dilution properties of successive batches of feed. This can be observed as a reduction of detection of PEDV RNA as subsequent batches of feed are manufactured [3], while still being able to detect contamination on environmental surfaces. These findings are hugely problematic because they describe that if PEDV enters a feed mill environment, the risk of transmission is not just with one batch of feed containing a contaminated ingredient, but that risk may persist across multiple batches of feed, including those that do not directly contain the suspect ingredient. Prior to this experiment, it was not known whether ASFV would have similar characteristics to PEDV and if biosecurity measures in place to detect PEDV would also be effective for ASFV detection within a feed manufacturing environment.

In the current experiment, it is evident that distribution of ASFV into the feed manufacturing environment is widespread and persists even after manufacturing additional feed batches initially free of ASFV. This is similar to what is observed with PEDV [3]. This indicates that it is extremely important for the US to prevent the entry of ASFV into US feed mills since once ASFV is in a feed mill, it will remain in its environment for an extended period of time. This knowledge is important to consider when designing and implementing surveillance and monitoring programs for ASFV as currently being investigated in ASFV endemic regions [15].

The present study demonstrates that transient surfaces had the highest amount of detectable ASFV DNA across all zones. This indicates that people and personal protective equipment (PPE) have a high potential to spread viruses within the feed mill. This is a consistent finding because it was previously reported that moving objects of a farm, like trucks and feed, contributed to the spread of PEDV, and that PPE and people transmitted PEDV to naïve herds [23, 24]. An understanding of the contamination within the feed mill environment is vital due to how the US manufactures and distributes feed within the swine industry. If a feed truck is contaminated, there is a risk that it could contaminate the production site it is delivering to, but it also could potentially contaminate the feed mill when returning from a production site currently experiencing a disease outbreak. Additionally, recent information from Vietnam has indicated that feed trucks are an area where contamination with ASFV can be found [15]. This current study along with previous studies highlight the importance of understanding the epidemiological interaction of the US feed delivery system regardless of the virus of concern.

A significant limitation of this study is the lack of infectivity data associated with the feed containing qPCR detectable ASFV-specific DNA. This research utilizes ASFV, a BSL-3 pathogen and select agent in the US, for which there are no validated virus isolation or pig bioassay methods. Validating these infection assays for feed are critically important, but out of the scope of this research. Our primary goal was to evaluate how the manufacture of feed with an ASFV-contaminated ingredient impacts the spread of that contamination throughout subsequent feed batches and the feed mill environment, which we have demonstrated with the response criteria selected in this study. We believe that the data herein provide significant value to the literature through establishing distribution characteristics of ASFV within a feed manufacturing facility which can provide critical background knowledge to assist with epidemiological investigations.

In conclusion, this study reveals that contamination with ASFV was rapid and widespread within the swine feed manufacturing facility after introduction through inoculated feed and presence of ASFV-specific DNA minimally changed with each subsequent batch. This study also proved that if there is viral contamination within the feed mill environment, it can be found with environmental swabs. In areas where ASFV is considered endemic, environmental swabs can be incorporated into surveillance programs or feed mill audits to understand the potential contamination within the feed mill and respective delivery system. In the present study, it was also demonstrated that transient surfaces play an important role in the spread of virus through the feed mill. Moving objects like people, PPE, and trucks should be taken in account when designing feed biosecurity protocols and feed/feed mill surveillance could be pivotal in maintaining appropriate feed biosecurity.

## Supporting information

S1 File Data file used for statistical analysis. (XLSX)
